# Prestat’s Adhesive Plaster

**Published:** 1845-01

**Authors:** 


*Prestat's Adhesive Plaster.*—The following composition is said never to crack, and not to inflame the skin:—Empl. diachyl. gum, 400 grains; purified rosin, 50 grains; tereb. venet., 38 grains, are mixed together at a gentle heat, and then 12 grains of gum mastich and 12 grains of gum ammoniac incorporated, and the mass spread on linen. In winter it is advisable to add 10 grains more turpentine, and 12 grs. ol. amygdal.—*Journ. für Prakt. Chem.*

